# *De novo* RNA sequencing analysis of *Aeluropus littoralis* halophyte plant under salinity stress

**DOI:** 10.1038/s41598-020-65947-5

**Published:** 2020-06-04

**Authors:** Elham Younesi-Melerdi, Ghorban-Ali Nematzadeh, Ali Pakdin-Parizi, Mohammad Reza Bakhtiarizadeh, Seyed Abolfazl Motahari

**Affiliations:** 1 Genetics and Agricultural Biotechnology Institute of Tabarestan, Sari Agricultural Sciences and Natural Resources University, Sari, Iran; 2 Department of Agronomy, Sari Agricultural Sciences and Natural Resources University, Sari, Iran; 3 Department of Animal and Poultry Science, College of Aburaihan, University of Tehran, Tehran, Iran; 4 Department of Computer Engineering, Sharif University of Technology, Tehran, Iran

**Keywords:** Bioinformatics, Agricultural genetics, Agricultural genetics

## Abstract

The study of salt tolerance mechanisms in halophyte plants can provide valuable information for crop breeding and plant engineering programs. The aim of the present study was to investigate whole transcriptome analysis of *Aeluropus littoralis* in response to salinity stress (200 and 400 mM NaCl) by *de novo* RNA-sequencing. To assemble the transcriptome, Trinity v2.4.0 and Bridger tools, were comparatively used with two k-mer sizes (25 and 32 bp). The *de novo* assembled transcriptome by Bridger (k-mer 32) was chosen as final assembly for subsequent analysis. In general, 103290 transcripts were obtained. The differential expression analysis (log_2_^FC^ > 1 and FDR < 0.01) showed that 1861 transcripts expressed differentially, including169 up and 316 down-regulated transcripts in 200 mM NaCl treatment and 1035 up and 430 down-regulated transcripts in 400 mM NaCl treatment compared to control. In addition, 89 transcripts were common in both treatments. The most important over-represented terms in the GO analysis of differentially expressed genes (FDR < 0.05) were chitin response, response to abscisic acid, and regulation of jasmonic acid mediated signaling pathway under 400 mM NaCl treatment and cell cycle, cell division, and mitotic cell cycle process under 200 mM treatment. In addition, the phosphatidylcholine biosynthetic process term was common in both salt treatments. Interestingly, under 400 mM salt treatment, the PRC1 complex that contributes to chromatin remodeling was also enriched along with vacuole as a general salinity stress responsive cell component. Among enriched pathways, the MAPK signaling pathway (ko04016) and phytohormone signal transduction (ko04075) were significantly enriched in 400 mM NaCl treatment, whereas DNA replication (ko03032) was the only pathway that significantly enriched in 200 mM NaCl treatment. Finally, our findings indicate the salt-concentration depended responses of *A. littoralis*, which well-known salinity stress-related pathways are induced in 400 mM NaCl, while less considered pathways, e.g. cell cycle and DNA replication, are highlighted under 200 mM NaCl treatment.

## Introduction

Soil salinity is an important challenge for food security and sustainable agriculture in the world^1^. Salt stress negatively influences the growth, development, and yield of agricultural products^2–4^. Higher plants adapt to different environmental stresses by complex molecular, cellular, and physiological mechanisms. The transcriptional alteration of stress-related genes is one of the key approaches at molecular level to cope with adverse conditions^5,6^. Plants employ complicated strategies such as ion homeostasis, osmotic regulation, antioxidant systems, and signaling pathways of phytohormones and MAPK to tolerate salinity stress^6^. Although our knowledge on this area is increasing continuously, but the molecular basis of salt resistance is not still well understood. Various omics technologies such as transcriptomics, proteomics, genomics and metabolomics are used for investigation of plant molecular responses^7,8^. Amongst these techniques, transcriptomics provides comprehensive insights into salt tolerance mechanisms in glyo- and halophyte plants. RNA sequencing (RNA-seq) is an accurate and high-throughput technique, which is widely used in transcriptome analysis studies^9,10^. Based on the availability of reference genome, *de novo* and genome-guided RNA-seq are used to evaluate the transcriptome. *De novo* RNA-seq is an innovative technique typically used for non-model plants that their genome is not available for transcriptome reconstruction^10^.

Halophyte plants are adapted for living in extreme environments and constitute about 1% of the Earth’s flora^3^. These plants are ideal candidates for studying and exploring the complex physiological and molecular mechanisms of salt resistance^11^. *Aeluropus littoralis*, a halophyte plant, is native to Iran and belongs to Poaceae family (grasses). This plant has C4 photosynthetic system and tolerates salinity up to 600 mM^12^. The small genome size, 342 Mb, and high salt tolerance ability of *A. littoralis*, make it a suitable candidate for salinity studies in monocotyledons. The transcriptome analysis of *A. littoralis* in different salt stress conditions can extend the molecular knowledge and identifying undercover mechanisms of salt resistance in halophytes. These findings can be subsequently used for improving economically important crop plants in genetic engineering and plant breeding programs. This study is the first report of high-throughput analysis (*de novo* RNA-Seq) of *A. littoralis* transcriptome that provides new insights at molecular responses of halophytes to salinity.

## Results

### Biochemical assessments

**Figure 1:**
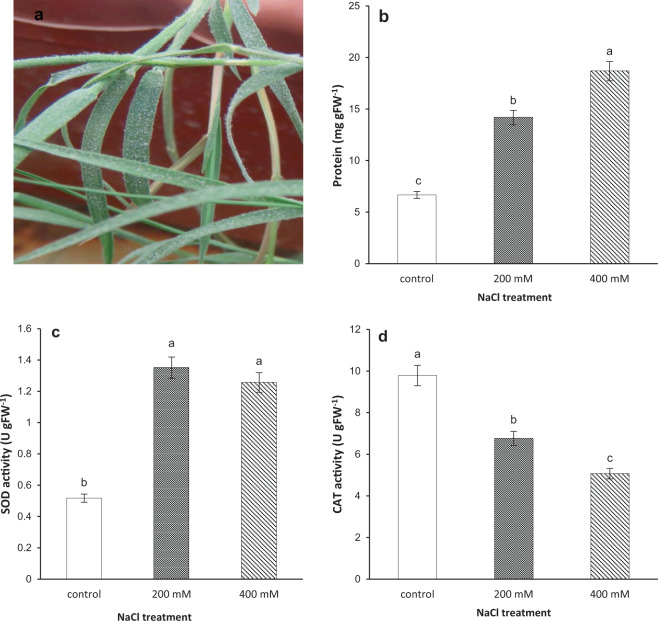
The effect of salinity on *A. littoralis* after 72 h of NaCl treatment (0, 200 and 400 mM) under hydroponic condition. (**a**) salt crystals on the leaf surfaces of *A. littoralis*, (**b**) leaf protein content of treated plants, (**c**) SOD enzyme activity and, (**d**) CAT enzyme activity of plant leaf samples. Data are presented as the mean of three biological replicates with error bars indicating SEM (n = 3). Different letters indicate significant differences at P ≤ 0.05.

After applying salt stress, the accumulation of salt crystals on the leaf surfaces of *A. littoralis* was apparently observed in both treatments (Fig. 1a). The variations in protein content and enzyme activities of salt treated samples in comparison with control plants clearly confirmed the incidence of salt stress. The results showed that protein content in both 200 and 400 mM NaCl treatments was significantly increased compared to control as well as compared to each other (Fig. 1b). Moreover, the SOD enzyme activity was considerably increased compared to control, however, no significant difference was observed between two salt treatments (Fig. 1c). In contrast, CAT enzyme activity was significantly decreased in both salt treatments compared to control and each other (Fig. 1d).

### Na^+^ and K^+^ concentrations

**Figure 2:**
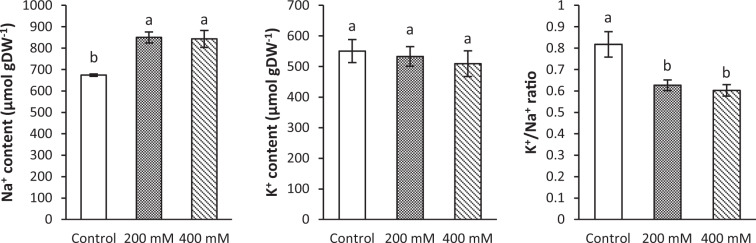
The Na^+^ and K^+^ contents and the K^+^/Na^+^ ratio in leaf samples of *A. littoralis* treated with different NaCl concentrations (0, 200 and 400 mM) after 72 h. Data are presented as the mean of three biological replicates with error bars indicating SEM (n = 3). Different letters indicate significant differences at P ≤ 0.05.

The Na^+^ concentration in *A. littoralis* leaves considerably increased in 200 and 400 mM NaCl treatments compared to the control (P < 0.05) after 72 h. However, the K^+^ content of leaf tissues was remained almost the same and no significant effect of NaCl concentration was observed. NaCl treatment showed a drastic effect on the K^+^/Na^+^ ratio in the salt treated plants and the ratio was significantly lower than control plants, though the difference between 200 and 400 mM salt treated plants was not considerable (Fig. 2).

### **RNA sequencing and*de novo*transcriptome assembly**

For RNA sequencing, nine cDNA libraries from three treatments (0, 200 and 400 mM NaCl) were generated, and a total of 364937505 clean reads were assembled using Trinity and Bridger tools with 25 and 32 k-mer sizes. The detailed information of the read numbers in different samples is provided in Supplementary File 2.

The results of assembly methods comparison showed that the 32 k-mer size is better in both assemblers. Summary of assembly statistics and BUSCO results are shown in Table 1 and Supplementary File 1 (Fig. S1), respectively. Assembly quality metrics revealed that Bridger (k-mer 32) transcriptome is the most appropriate, with the largest N50 value (2076). In addition, assembly completeness evaluation by BUSCO showed better results for Bridger (k-mer 32) transcriptome and 88.1% of 1440 conserved plant genes were present in the assembled contigs. Whereas, Trinity (k-mer 32) assembled transcriptome includes 85.2% of the conserved plant-specific orthologous genes. Therefore, the *de novo* transcriptome generated by Bridger (k-mer 32) was selected for subsequent analysis. This transcriptome includes 103290 transcripts (isoforms) and 83344 genes with a GC content of 49.72%. Length distribution of assembled contigs by Bridger (k-mer 32) is shown in Supplementary File1 (Fig. S2). By considering transcript expression, 17487 transcripts represented 90% of the total expression data (Ex90) and had an N50 of 2254 bp (Ex90N50).

**Table 1:** Statistics summary of four different *de novo* transcriptome assembly strategies.

Software	K-mer length	N50 length	Mean length	Median length	Number of assembled bases
Bridger	32	2076	1110.58	578	114459684
	25	2014	1110.55	596	114712063
Trinity	32	1789	1098.75	732	182200148
	25	1693	1045.16	692	173634303

### Functional annotation of the *A. littoralis* transcriptome

Selected assembled transcripts were annotated by different tools (BLAST, HMMER, TransDecoder, RNAMMER v1.2, TMHMM v2.0, SignalP v4.1, and KASS) and public databases (UniProtKB, Rfam, miRbase, Pfam, Rnammer, tmHMM, signalp, GO and KEGG). Candidate coding regions in *A. littoralis* transcriptome (103290 transcripts) were identified by TransDecoder and 142168 ORFs and 62586 probable coding sequences were predicted. Sequence homology search results against the UniprotKB/SwissProt database by BLASTP (E-value > 1e^*−*5^, for predicted protein sequences) and BLASTX (E-value > 1e^*−*5^, for assembled transcripts) were 40809 (39.5%), and 42710 (41.34%) annotated contigs, respectively. The BLASTX against the uniprot_trEMBL_plants database revealed that *A. littoralis* transcripts have highest similarity with Poales and Poaceae family (92%). The similarity species distribution of Poaceae family was as *Arundo donax* (15%), *Setaria viridis* (12%), *Panicum miliaceum* (9%), *Zea mays* (7%), *Sorghum bicolor* (7%), *Setaria italica* (6%) and other Poaceae (14%) (Supplementary File1, Fig. S3).

A total of 155341 probable domains in *A. littoralis* transcriptome were identified by searching against Pfam database and, these domains were classified into 9775 unique domain groups. The four most abundant domain groups were protein kinase, protein tyrosine kinase, phosphotransferase enzyme family, and PPR repeat. The homology search against the miRBase database resulted in the annotation of 105 transcripts as potential miRNAs. In general, 53262 contigs (51.5% of all transcripts) annotated within at least one functional database. The Venn diagram of overlapping annotated transcripts in *A. littoralis* transcriptome against different databases is shown in Supplementary File 1 (Fig. S4). Detailed information on the annotations is represented in Supplementary File 3.

Functional annotation was followed by GO analysis and 41764 transcripts were categorized into 76 GO terms. The number of transcripts in three main categories of biological process (BP), molecular function (MF) and cellular component (CC) was 34385 (33.2%), 35949 (34.8%) and 36074 (34.9%), respectively. The most dominant GO term in the biological process category was “zinc ion binding,” with 12517 transcripts, followed by “sequence-specific DNA binding” (7015 transcripts) and “endonuclease activity” (4127 transcripts). In the molecular function category‌, “RNA modification” (13625 transcripts), “defense response” (8808 transcripts) and, “cell division” (3955 transcripts) were the most prominent. In the cellular component category, “mitochondria” (22119 transcripts), “plant-type cell wall” (5881 transcripts) and “integral component of membrane” (4596 transcripts) were the most abundant terms (Supplementary File 1 & Supplementary Files 4–6). KEGG enrichment analysis was performed to identify active metabolic processes in *A. littoralis* transcriptome. In conclusion, 17832 transcripts were assigned to 390 KEGG pathways and 42630 transcripts were assigned to at least one KEGG pathway or GO term (Supplementary File 3).

### PCA and differential expression analysis

**Figure 3:**
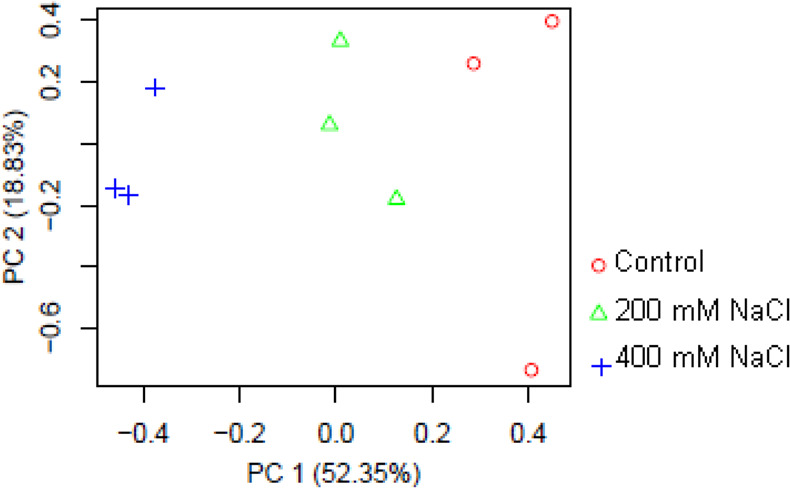
Principal component analysis (PCA) on the read counts of *A. littoralis* in response to different NaCl concentrations (0, 200 and 400 mM).

Results of PCA analysis revealed the distinct differences in transcript expression patterns among the treatments. As can be seen in Fig. 3, the first two principal components contain 71.18% of the information and the treatments are grouped in separate clusters that highlights particular response of *A. littoralis* to different salt concentrations. The first principal component contains 52.35% of the variance and the second principal component contains 18.83% of the variance.

For finding the differentially expressed transcripts, two pair-wise comparisons, 200 mM vs. control and 400 mM vs. control, were carried out (log_2_^FC^ > 1 and FDR < 0.01). A total of 485 differentially expressed transcripts were identified in the 200 mM vs. control comparison, including 169 up and 316 down-regulated transcripts. Moreover, 1465 differentially expressed transcripts were found in the pair-wise comparisons between 400 mM and control, including 1035 up-regulated and 430 down-regulated transcripts. In up-regulated transcripts (1204 transcripts), 89 transcripts were common in both salt treatments, 944 and 80 transcripts were specifically up-regulated in 400 and 200 mM salt treatments, respectively. On the other hand, in 746 down-regulated transcripts, 49 transcripts were common in both salt treatments, 381 and 265 transcripts were specifically down-regulated in 400 and 200 mM NaCl treatments, respectively. Interestingly, two transcripts with different expression patterns (up-regulation in 400 and down-regulation in 200 mM) were identified in both treatments. The detailed information of the differentially expressed transcripts in each of pair-wise comparisons is provided in Supplementary File 7.

By homology search of the differentially expressed transcripts against the PlantTFDB database, a total of 67 transcripts were annotated and categorized into 20 families. ERF and WRKY were the most abundant TF families with 24 and 9 transcripts, respectively. In 400 mM NaCl treatment, 48 TFs were up and 17 TFs were down-regulated, while in 200 mM salt treatment, 2 TFs were up and 4 TFs were down-regulated (Supplementary File 8).

### Functional enrichment of differentially expressed genes

To investigate the function of differentially expressed genes (DEGs), GO enrichment analysis was performed using goseq package. Among the 1035 up-regulated DEGs in 400 mM NaCl treatment, 514 genes were significantly assigned to 120 GO terms (FDR < 0.05). Amongst 68 GO terms in the biological process (BP), “chitin response” (child GO terms; response to abscisic acid, regulation of jasmonic acid mediated signaling pathway, stress-activated protein kinase signaling cascade, response to lipid, ethylene-activated signaling pathway and else), “polysaccharide catabolism” (child GO terms; phosphatidylcholine biosynthetic process, oxidation-reduction process, polysaccharide catabolic process, else), and “response to stimulates” were the most important terms. Furthermore, 50 GO terms were involved in molecular function (MF), in which “hydrolyzing O-glycosyl compounds” and “amylase activity” were the dominant categories. The “PRC1 cell complex” and “vacuole” terms were also enriched in cellular component (CC). Moreover, 145, 149, and 72 out of 430 down-regulated DEGs in 400 mM NaCl treatment were significantly assigned to 12 BP, 27 MF and 6 CC, respectively (FDR < 0.05).

Up-regulated DEGs in 200 mM NaCl treatment (169 DEGs) were grouped in 7 BP (including “cell cycle”, “cell division” and “mitotic cell cycle process”), 27 MF and 6 CC terms. Also, 2 MF and 2 CC terms in 200 mM NaCl treatment were enriched by 316 down-regulated DEGs, while no significant BP terms were found in this treatment (Supplementary File 9). The top five over-represented GO terms in BP, MF and CC categories (FDR < 0.05) are reported in Table 2.

**Table 2:** The top five significantly over-represented GO terms in up and down-regulated DEGs under 200 and 400 mM NaCl treatments (FDR < 0.05).

	400 mM NaCl		200 mM NaCl	
	Up-regulated DEGs	Down-regulated DEGs	Up-regulated DEGs	Down-regulated DEGs
Biological process (BP)	phosphatidylcholine biosynthetic process (GO:0006656)	hydrogen peroxide catabolic process (GO:0042744)	cell division (GO:0051301)	—
	choline biosynthetic process (GO:0042425)	reactive oxygen species metabolic process (GO:0072593)	microtubule-based movement (GO:0007018)	—
	response to chitin (GO:0010200)	cell wall organization or biogenesis (GO:0071554)	movement of cell or subcellular component (GO:0006928)	—
	response to oxygen-containing compound (GO:1901700)	external encapsulating structure organization (GO:0045229)	phosphatidylcholine biosynthetic process (GO:0006656)	—
	polysaccharide catabolic process (GO:0000272)	defense response (GO:0006952)	mitotic cell cycle process (GO:1903047)	—
Cell component (CC)	PRC1 complex (GO:0035102)	extracellular region (GO:0005576)	kinesin complex (GO:0005871)	kinetoplast (GO:0020023)
	plant-type vacuole (GO:0000325)	cell wall (GO:0005618)	microtubule associated complex (GO:0005875)	mitochondrial part (GO:0044429)
	—	external encapsulating structure (GO:0030312)	microtubule (GO:0005874)	—
	—	plant-type cell wall (GO:0009505)	cytoskeletal part (GO:0044430)	—
	—	anchored component of plasma membrane (GO:0046658)	DNA packaging complex (GO:0044815)	—
Molecular function (MF)	phosphoethanolamine N-methyltransferase activity (GO:0000234)	glucan endo-1,3-beta-D-glucosidase activity (GO:0042973)	microtubule motor activity (GO:0003777)	oxidoreductase activity, acting on single donors with incorporation of molecular oxygen (GO:0016701)
	hydrolase activity, hydrolyzing O-glycosyl compounds (GO:0004553)	tetrapyrrole binding (GO:0046906)	tubulin binding (GO:0015631)	oxidoreductase activity, acting on single donors with incorporation of molecular oxygen, incorporation of two atoms of oxygen (GO:0016702)
	oxidoreductase activity (GO:0016491)	peroxidase activity (GO:0004601)	motor activity (GO:0003774)	—
	hydrolase activity, acting on glycosyl bonds (GO:0016798)	heme binding (GO:0020037)	phosphoethanolamine N-methyltransferase activity (GO:0000234)	—
	amylase activity (GO:0016160)	oxidoreductase activity, acting on peroxide as acceptor (GO:0016684)	cytoskeletal protein binding (GO:0008092)	—

### KEGG enrichment

Generally, DEGs were assigned to five categories in KEGG database, pathways related to metabolism, genetic information processing, environmental information processing, cellular process, and organismal systems. Results of GOseq analysis revealed that in 200 mM NaCl treatment just one pathway, DNA replication proteins (ko03032), was significantly enriched in up-regulated genes, and no pathways were enriched in down-regulated genes. While, in 400 mM NaCl treatment, six and three pathways were significantly enriched with up and down-regulated genes, respectively (Table 3). Two pathways of plant MAPK signaling pathway (ko04016), and plant hormone signal transduction (ko04075) were significantly enriched in both up and down-regulated genes in 400 mM NaCl treatment.

**Table 3:** Significantly over-represented KEGG pathways with up and down-regulated genes under 200 and 400 mM NaCl treatments (FDR < 0.05).

	Ko id	Term	FDR
400 mM NaCl (up-regulated)	ko00500	Starch and sucrose metabolism	0.000125
	ko00199	Cytochrome P450	0.01816
	ko04016	MAPK signaling pathway - plant	0.01816
	ko04075	Plant hormone signal transduction	0.01816
	ko00460	Cyanoaminoacid metabolism	0.01816
	ko00908	Zeatin biosynthesis	0.01816
400 mM NaCl (down-regulated)	ko00940	Phenylpropanoid biosynthesis	0.000324
	ko04016	MAPK signaling pathway - plant	0.028518
	ko04075	Plant hormone signal transduction	0.028518
200 mM NaCl (up-regulated)	ko03032	DNA replication proteins	0.011084

### Phytohormone signal transduction

**Figure 4:**
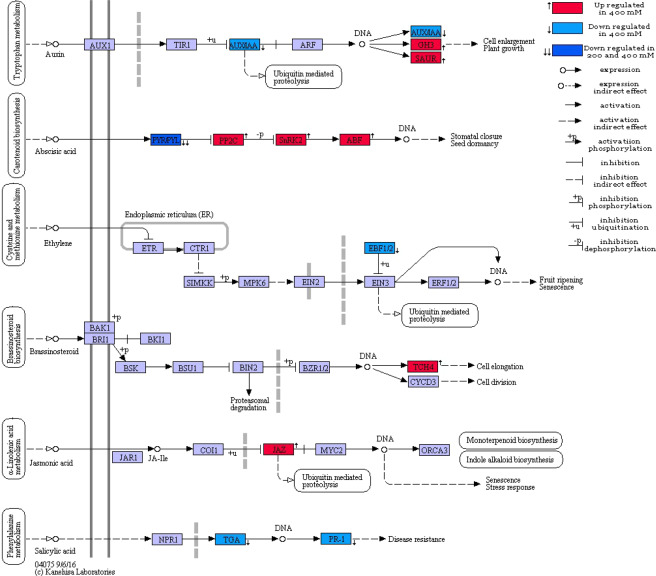
The effect of 200 and 400 mM NaCl treatment on phytohormone signal transduction pathway (Ko04075). Auxin-responsive protein IAA (*AUX*/*IAA*), auxin responsive GH3 gene family (*GH3*), SAUR family protein (*SAUR*), abscisic acid receptor PYR/PYL family (*PYR-PYL*), protein phosphatase 2 C (*PP2C*), serine/threonine-protein kinase SRK2 (*SNRK2*), ABA-responsive element binding factor (*ABF*), EIN3-binding F-box protein (*EBF1_2*), xyloglucan: xyloglucosyl transferase TCH4 (*TCH4*), jasmonate ZIM domain-containing protein (*JAZ*), transcription factor TGA (*TGA*) and pathogenesis-related protein 1 (*PR-1*). Plant Hormone Signal Transduction Pathway adapted from KEGG^77^.

This pathway was significantly enriched in both up and down-regulated genes in 400 mM NaCl treatment. Moreover, our results showed that the signaling pathway of most plant hormones including auxin, gibberellin, abscisic acid, ethylene, jasmonic acid, brassinosteroid, and salicylic acid was affected by the treatment of 400 mM NaCl treatment. In this regard, the *PYL* gene was down-regulated and *PP2C*, *ABF* and, *SNRK2* genes up-regulated in the ABA signaling pathway (Fig. 4). In addition, in the signaling pathway of auxin hormone, all three classes of primary auxin-response genes (*IAA*, *GH3* and *SAUR*) were strongly affected by 400 mM NaCl. Hence, *GH3* and *SAUR* genes were over-expressed and *IAA* gene was down-regulated. Also, the down-regulation of *IAA* and *PYL* genes were observed in 200 mM NaCl treatment (Fig. 4). For additional details see Supplementary File 10.

### MAPK signaling pathway

**Figure 5:**
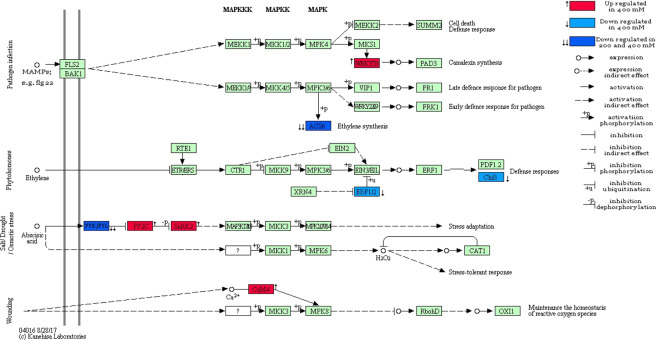
The effect of 200 and 400 mM NaCl on plant MAPK signaling pathway (Ko04016). WRKY transcription factor 33 (*WRKY33*), 1-aminocyclopropane-1-carboxylate synthase 1/2/6 (*ACS6*), abscisic acid receptor PYR/PYL family (*PYR-PYL*), protein phosphatase 2 C (*PP2C*), EIN3-binding F-box protein (*EBF1_2*), basic endochitinase B (*ChiB*) and calmodulin (*CaM4*). MAPK Signaling Pathway-Plant adapted from KEGG^77^.

Another important pathway of message transduction, which significantly enriched in 400 mM treatments with DEGs (up and down-regulated) was MAPK signaling pathway. Whereas the MAPK signaling pathway was not significantly enriched in 200 mM treatment, but two genes (*ACS6* and *PYL*) were significantly down-regulated in the pathway (Fig. 5). The *ACS6* showed the highest down-regulation (238.85 fold, log_2_^FC^ = −7.9) in both 200 and 400 mM NaCl treatments. On the other hand, the *PP2C* (protein phosphatase 2C) showed the highest up-regulation (32 fold, log_2_^FC^ = 5) in the 400 mM NaCl treatment (Supplementary File 10).

### DNA replication

In the 200 mM NaCl treatment, only DNA replication-associated proteins with up-regulated genes were significantly enriched. However, no pathway was found for the down-regulated genes. Subunit I DNA polymerase epsilon, factor MCM3 of helicase, the large subunit of DNA primase, and replication factor A1 of RPA were significantly up-regulated. Subunit I DNA polymerase epsilon was the only component that was identified in both salt treatments (Supplementary File 10). In the 400 mM NaCl treatment, no transcripts related to DNA replication pathway were significantly enriched.

### Validation of DEGs by qRT-PCR

**Figure 6:**
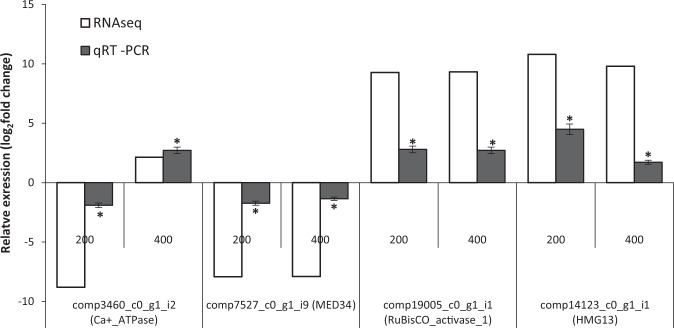
qRT-PCR validation of RNA-seq data. Four differentially expressed genes in the RNA-seq experiment were analyzed by qRT-PCR. Data are shown as the fold change at 200 and 400 mM NaCl relative to the control (No NaCl). The expression data were normalized to *gapdh* as a reference gene. Relative expression was calculated based on the 2^−ΔΔCt^ method and represent the mean expression value ± SEM (n = 3) relative to the control. (*) represents a statistically significant difference (P ≤ 0.05) when compared to the control.

To validate RNA-seq results, four differentially expressed transcripts in the RNA-seq experiment were randomly selected for qRT-PCR. The results showed that the DEGs selected for validation exhibited similar up or down-regulation patterns as those shown from RNA-seq data analysis (Fig. 6), although the observed relative expression differences between RNA-seq and qRT-PCR is owing to intrinsic features of these methods.

## Discussion

Salt stress affects various biochemical, physiological and molecular processes in plants. The halophytes, such as *A. littoralis*, have a variety of well-adapted mechanisms to encounter with the saline conditions and mitigate the adverse effects of salinity on the plant growth and development.

It is thought that Na^+^ and K^+^ homeostasis is crucial for salt-tolerance in plants^13^. In our study the Na^+^ content of *A. littoralis* leaves at 200 and 400 mM NaCl increased in comparison with the control plants. However, the Na^+^ concentration difference in 200 and 400 mM salt treated plants was not significant. This could be the result of excretion of elevated levels of Na^+^ from the salt glands on the plant leaf surface. Similarly, accumulation of Na^+^ in the shoots and roots of *A. littoralis*^14,15^ and *A. lagopoides*^16^ in different salt stress treatments has been reported. It has shown that halophyte grasses like *Aeluropus* spp. may employ more efficient mechanisms for K^+^ homeostasis and Na^+^ exclusion than ion sequestration mechanisms generally used by dicot halophytes^17,18^.

Study of the salt tolerance mechanisms in halophyte plants provides valuable information for designing crop breeding programs. Besides, *de novo* transcriptome assembly method offers a notable opportunity to reconstruct reference transcriptome in non-model organisms without reference genome sequence as well as allows the discovery of novel genes. In the present study, the transcriptional response of *A. littoralis*, a halophyte plant (Poaceae), was evaluated under two salt concentrations in 72 h after stress using *de novo* RNA-seq analysis. To reconstruct the reference transcriptome, two assemblers including Trinity and Bridger, which are the most reliable and widely used assembly tools, were chosen^19–21^. The Trinity package and Bridger have already been used to assemble the transcriptome of several plant species^21–23^. The key idea of Bridger’s algorithm is to create a bridge between the reference genome-based assembly tools (e.g. Cufflinks) and those which assemble without the reference genome (e.g. Trinity)^24^. The results of this study showed that the contigs obtained from Bridger with a length of 32 k-mer provides better data for performing downstream analyses. Although Trinity had more assembled bases than Bridger, length-related statistics indicated the higher quality of Bridger in both k-mers. Furthermore, results of BUSCO analysis showed the superiority of Bridger to Trinity, which is in agreement with previous studies (Table 1 and Supplementary File 1 (Fig. S1))^25,26^. Therefore, the *de novo* assembled transcriptome by Bridger (32 k-mer) was used for further analysis. The GC content of the reference transcriptome was 49.72%, which is in the range of previous studies in *Festuca arundinacea* (49.49%) and *Arundo donax* (49%) transcriptomes (both belongs to Poaceae family)^27,28^.

To provide a biological interpretation of assembly results, the differential expression analysis and functional annotation of 103290 transcripts were done. Results of GO enrichment of DEGs revealed that the phosphatidylcholine biosynthetic process was the only common term between two salt treatments in BP category. Accordingly, most of the well-known terms related to stress such as oxidation-reduction process, ROS metabolic process, response to abscisic acid and regulation of jasmonic acid mediated signaling pathway were induced under 400 mM NaCl treatment. In contrast, cellular-related biological processes like cell cycle and cell division were induced under 200 mM NaCl (Supplementary File 9). These results indicated that *A. littoralis* responses to 200 and 400 mM NaCl in the completely different manners. Hence, it can be stated that 200 mM NaCl is not detected as stress condition, but 400 mM NaCl induces the most stress-related terms in the *A. littoralis*. Our results support previous findings that 200 mM NaCl enhances the biomass of *A. littoralis* in liquid MS culture medium^29^ and also increases the root length in comparison with control plants^30^.

Phosphatidylcholine is a dominant phospholipid in the cell membrane and forms 40 to 60% of membrane lipids^31^. It seems that in higher plants this compound, due to its role in maintaining the cell membrane integrity, is a major contributor to the adaptation response to environmental stresses^32^. In the present study, two transcripts encoding phosphoethanolamine N-methyl transferase (*NMT*, EC:2.1.1.103) and choline-phosphate cytidylyl transferase (*PCYT1*, EC:2.7.7.15) were up-regulated under both salt treatments in the phosphatidylcholine biosynthetic process. Up-regulation of these genes under salt stress was also reported in the comprehensive transcriptome analysis of *Sporobolus virginicus* halophyte plant^33^. Mou *et al*. (2002) reported that inactivation of *NMT* gene in Arabidopsis caused a high sensitivity to salt stress, therefore they suggested that this enzyme plays an important role in the salt stress tolerance^34^.

Interestingly, our results indicated that chromatin modification plays an important role in the response to salinity stress in *A. littoralis*. In this regard, the PRC1 complex which plays a major role in chromatin remodeling was enriched in up-regulated genes in 400 mM NaCl treatment (Supplementary File 9). In *Drosophila melanogaster*, as a model organism in chromatin studies, the PRC1 complex mediates the ubiquitination of the lysine residue in histone H2A and is involved in maintaining long-term transcriptional suppression and plays role in the epigenetic alteration of chromatin remodeling^35^. It has long been thought that PRC1 complex is absent in plants, but this opinion was changed by the discovery of the ubiquitinated residue of H2A histone in *A. thaliana*. In plants, PRC1 complex has five subunits and is related to gene deactivation^36^. Furthermore, other evidences such as the presence of nucleosome/chromatin assembly factor group D13 (*HMGB13*) and structural maintenance of chromosomes 4 (*SMC4*) genes in top 20 over-expressed genes in both salt treatments (Supplementary File 7) directed us to conclude about the importance of chromatin modification in response to salinity stress. HMGBs involves in DNA-related cellular processes such as duplication, transcription, and chromatin assembly^37^. The Damage-associated molecular pattern-like activities of *HMGB3* was reported in Arabidopsis^36^. Besides, expression changes of some members of the HMG family have been reported in response to abiotic and biotic stresses in Arabidopsis^38,39^. The *SMC4* is one of the catalytic members of the chromatin condensing complex which participates in the chromatin condensation during mitosis and chromosome segregation in meiosis^38^. It has been reported that *SMC4* has an important role in the formation of Arabidopsis epigenome and collaborates with DNA methylation to assemble suppressor chromatin complexes^40^. Different expression pattern of *ALP1* (protein-antagonist of like heterochromatin protein 1), another gene that involves in chromatin remodeling, was observed in 200 mM (log_2_^FC^ = −8.72) and 400 mM (log_2_^FC^ = 3.6) NaCl treatments (Supplementary File 7). The role of *ALP1* in opposing PcG protein-mediated chromatin silencing in Arabidopsis has been reported^41^. Altogether, these results indicated that the mentioned pathways and genes may have led to chromatin modification in response to salinity stress in *A. littoralis*.

Plants as immobile organisms are constantly subjected to the environmental stimuli, so they need an efficient and effective signaling system to response to these stimuli. Based on the KEGG enrichment results, the plant MAPK signaling and phytohormone signaling pathways were enriched with both up and down-regulated transcripts under 400 mM NaCl treatment. Although, these pathways were not enriched under 200 mM NaCl treatment, but some transcripts including *IAA*, *PYL*, and *ACS6* were showed notable differential expression compared to control (Fig. 4 and 5).

MAPK cascades are known as a key signaling pathway involved in response to various stresses^42,43^. Although in our results, kinase proteins of this pathway were not detected, but some of downstream genes such as *WRKY33* showed differential expression after 72 h salt stress. Expression of *WRKY33‌* transcription factor was up-regulated in 400 mM NaCl treatment, and no change was observed in 200 mM treatment. Salt stress sensitivity has been increased in wrky33 null and wrky25wrky33 double mutants of Arabidopsis. However, overexpression of *PcWRKY33* (*Polygonum cuspidatum)* has decreased salt stress tolerance in Arabidopsis^42^.

Phytohormones play an important role in regulation of plant response to different stresses. In our study, signaling pathways of the abscisic acid (ABA), auxin, ethylene, Brassinosteroids, jasmonic acid, and salicylic acid were affected by 400 mM NaCl treatment.

Stimulation of plant hormone signaling pathways is shown in response to salinity stress^44^. ABA is known as stress hormone and plays a critical role in response to various stresses^45,46^. All key genes of ABA signaling pathway, including *PYL* and *PP2C*, were induced under 400 mM salt treatment, but only *PYL* was affected by 200 mM NaCl treatment. *PYL* was down-regulated in both salt treatments, while *PP2C* was up-regulated in 400 mM NaCl treatment (Fig. 4 & Supplementary File 10). Our results showed that up-regulation of both *PYL* and *PP2C* genes is not crucially necessary for steadiness of ABA signaling pathway, that is in consistent with previous findings^46^.

Auxin signaling genes classified into three main classes including auxin response genes, auxin response factors, and primary auxin-response genes (including *IAA*, *GH3* and *SAUR*). Our results showed that all three primary auxin-response genes were strongly affected by 400 mM NaCl (Fig. 4 & Supplementary File 10). In several studies up-regulation of various members of the GH3 family in sorghum, chickpea, medicago and rice under salt and drought stress is reported^47–49^. For instance, it has shown that the majority of *OsGH3* genes, namely *OsGH3-2* and *OsGH3-8* were up-regulated in rice under salt stress^49^.

The DNA replication pathway was the only enriched pathway in 200 mM treatment, and all related genes (including *PRI2*, *POLA*, *MCM3*, and *RAF1*) were up-regulated. To the best of our knowledge, the reports on the effect of salt treatments on the genes involved in DNA replication pathway are limited. It has been shown that a component of the *MCM2–7* complex may function as a DNA helicase which is necessary for replication initiation and elongation per cell cycle in eukaryotic cells^50^. Transcriptome analysis of seagrass *Zostera marina* L. by RNA-seq showed that DNA replication pathway was significantly enriched by up-regulated genes such as the *MCM6* gene. The enhanced salinity stress tolerance in tobacco plants due to overexpression of *MCM6* has been reported. In addition, it is shown that promoter region of this gene contains responsive elements to salinity and cold stresses^51^.

Among salinity stress-responsive genes, TFs play a key role in regulating the molecular responses at the transcriptional level^50^. In this study, 67 differentially expressed TFs were found within 20 different families, in which the *ERF*, *WRKY*, *C2H2*, *NAC*, *bZIP*, *MYB*-related and *MYB* were the largest families (Supplementary File 8). Previous studies in plants have been reported the response of same TF families to the salt stress^52,53^. The comparison between the two salt treatments showed that the number of families and their members in the 400 mM treatment was remarkably higher than 200 mM treatment. Most of the differentially expressed TFs in both treatments were related to ERF family. In this regard, 16 and 7 members of this family were up and down-regulated in the 400 mM NaCl treatment, respectively. In 200 mM NaCl treatment, all four down-regulated TFs were belongs to ERF family (Supplementary File 8). It is well known that the activation of a large number of stress-responsive genes is mediated through specific TFs, which play important roles in the regulation of secondary metabolites biosynthesis in plants. The role of ERF family in initiation of appropriate response to salt stress has been confirmed in previous studies^54–56^. Significant induction of *ERF76* gene has been reported in the leaves, roots, and stems of *Populus simonii* × *Populus nigra* under salt stress. Overexpression of the *ERF76* gene increased the tolerance of transgenic poplar to salt stress^55^, indicating that ERF family could be considered as an important family of TFs in response to stress. Additionally, members of GRF family (lesser-known in salt stress), was identified in 200 mM NaCl treatment (Supplementary File 8). In contrast to the ERF, GRF is a small family of plant-specific TFs that involves in growth, development, and reproduction and differentiation processes^57^. Recently a few studies reported the role of this family in coordination plant growth in abiotic and biotic stress conditions^57–59^. For example, seven genes of GRFs family were differentially expressed in the tomato leaves at different time points under NaCl stress^57^.

It should be noted that a little proportion of up and down-regulated transcripts in 400 mM and up-regulated transcripts in 200 mM salt treatment were categorized as unknown function (NA) transcripts. On the other hand, more than 50% of down-regulated transcripts in 200 mM NaCl treatment with high expression value categorized as NA (Supplementary File 7).

## Conclusion

In this study the *A. littoralis* responses to salinity stress at transcriptome level were investigated by *de novo* RNA-seq analysis. Our findings highlighted the well-known salt tolerance mechanisms such as MAPK and phytohormones signaling, synthesis of osmolytes, and ionic homeostasis under 400 mM NaCl treatment. In contrast, the processes related to growth such as cell division and DNA replication were enriched in GO and KEGG analysis by up-regulated transcripts under 200 mM NaCl treatment. Several pieces of evidence supported the key role of chromatin remodeling in response to salinity stress in *A. littoralis*. Finally, our findings suggest that *A. littoralis* responses to salt stress are greatly salt concentration dependent. It seems likely that 200 mM NaCl is not serves as stress condition and actually enhances the plant growth, but 400 mM NaCl induces plant tolerance mechanisms. This study has gone some way towards increasing our understanding of salt tolerance molecular mechanisms in halophyte plants. Future studies will focus on lesser-known processes and pathways and their relation with salinity tolerance in *A. littoralis*.

## Materials and Methods

### Plant material

The *A. littoralis* seedlings were grown under hydroponic culture in Hoagland solution^60^ (pH = 5.8) and greenhouse condition (photoperiod 16 h light and 8 h darkness, 27 °C and 65% relative humidity). To minimize the possible genetic variation of plant material, a single plant was propagated by successive cutting. After obtaining adequate plant materials, rooted cuttings were grown in the pots. Aeration was carried out using air pumps, and Hoagland solution was replaced with fresh solution every two weeks. The one-month seedlings were subjected to different concentrations of NaCl (200 and 400 mM). After 72 h of salt treatment, leaf, stem, and root samples were harvested from the control (0 mM NaCl) and salt-treated plants and were stored in −80 °C.

### Preparation of protein extract

For preparing the plant protein extract, 200 mg of fresh leaf samples were homogenized with 1800 μl of 50 mM potassium phosphate buffer (pH 7.0). The homogenates were centrifuged at 20000×g for 20 min at 4 °C. The supernatant was dispensed in aliquots and stored at −80 °C for subsequent protein content determination and enzyme activity assays.

### Protein content measurement

Protein content of leaf samples (three replicates) was determined according to the method of Bradford^61^. Briefly, 200 μl of protein extract, 1800 μl of distilled water and 500 μl of Bradford solution were mixed and the absorbance was recorded at 595 nm. Protein concentration is calculated from standard curve of Bovine Serum Albumin (BSA).

### SOD enzyme activity assay

The activity of SOD enzyme was measured by the evaluation of its inhibitory effect on nitrobluetetrazolium (NBT)^62^. The 2 ml assay reaction mixture contained phosphate buffer (50 mM), methionine (0.013 mM), EDTA (0.1 μM) and riboflavin (2 μM). Immediately after adding riboflavin, reaction mixture was poured into the test tube containing 50 μl of protein extract and exposed to the light for 15 min. The absorbance of samples was measured at 560 nm. The activity of SOD enzyme was calculated based on the enzyme unit per g fresh weight (U gFW^−1^).

### Catalase enzyme activity assay

CAT enzyme activity was determined by measuring the decrease in absorbance at 240 nm per unit time due to decomposition of H_2_O_2_. The reaction mixture consisted of 1000 μl of 100 mM phosphate buffer (pH = 7) and 750 µl of 15 mM H_2_O_2_. The reaction was started by adding 75 μl of the enzyme extract. The enzyme activity was expressed as enzyme unit per g fresh weight (U gFW^−1^)^63^.

### Na^+^ and K^+^ determination

The plant leaves were thoroughly rinsed with deionized water and then dried at 80 °C for 72 h. 500 mg of dried leaf samples were kept in a furnace at 550 °C for 6 h and the resulting ash was extracted with 5 ml 2 N HCl for 30 min. The solutions were diluted with deionized water and after filtration, the amount of Na^+^ and K^+^ was determined with a PFP7 Flame Photometer, GENWAY, UK.

### Statistical Analysis

Effect of different salinity treatments on protein content, the SOD, and catalase enzymes activity were analyzed using the GLM procedure of SAS statistical software version 9.1 according to the following statistical model:

Y_ij_ = μ + T_i_ + e_ij_

Which, Y_ij_ is the E-value of the studied trait, μ is mean of studied trait, T_i_ is the effect of treatments and e_ij_ are random errors. The mean of the treatments was compared using Duncan’s multiple range test.

### RNA extraction and Illumina sequencing

Total RNA was isolated using RNeasy Plant Mini Kit (Qiagen) according to the manufacturer’s protocol and treated with DNase I (Fermentas). RNA quantity and quality were evaluated using spectrophotometry, Agilent 2100 Bioanalyzer and 1% agarose gel. RNA samples with a ratio of 260/280 nm and 28 S/18 S greater than 1.8 and RNA integrity number (RIN) > 8 were selected for subsequent processes. Subsequently, nine cDNA libraries were constructed and each cDNA library was sequenced using the Illumina HiSeq 2500 platform to generate 150 bp paired-end reads.

### Quality control and *de novo* assembly

The quality of the raw reads was evaluated using the FastQC (http://www.bioinformatics.babraham.ac.uk/projects/fastqc/)^64^ and the low-quality bases (<20) and adapter contamination were removed using Trimmomatic v0.38^65^. The trimmed data were again evaluated by FastQC to confirm the quality of reads.

To obtain the most appropriate *de novo* transcriptome assembled results and increase the confidence of data quality, two different transcriptome assembly approaches including Trinity v2.4.0^22,66^ and Bridger^24^ were compared. Each assembler was run with two k-mer sizes (25 and 32 base pair). While Trinity employs the De Bruijn method to perform assembly^25,26^, Bridger applies a rigorous mathematical model to construct splicing graphs^27^. Following, the four contig datasets, Trinity (25-mer), Trinity (32-mer), Bridger (25-mer) and Bridger (32-mer) reassembled by Cap3 tool^67^ to perform a meta-assembly, reducing redundancy and generating more complete consensus sequences (cutoff 95%). Finally, the results were evaluated by different quality metrics including N50 length of the transcriptome assemblies, the total number of bps in the assembly and BUSCO v2.0.1 analysis using OrthoDB v9.1 ‘embryophyta’ database as a reference to assess the assembly and annotation completeness^68^.

### Functional annotation

The best-assembled transcriptome was annotated using several approaches. First, TransDecoder tool^69^ was used to find the probable coding regions of transcripts and the open reading frames (ORFs) with a minimum length of 100 amino acids. Then, functional annotation of the transcripts was performed using BLASTX against UniProtKB/SwissProt databases and uniprot_trEMBL_plants database (E-value <1e^−5^). Moreover, a homology search based on the BLASTP was performed using the predicted proteins as query against UniProtKB/SwissProt databases (E-value <1e^−5^). To recognize homologous non-coding RNAs as well as miRNAs, transcript sequences were aligned against the miRbase and Rfam databases using BLASTN tool (E-value <1e^−5^). To determine the protein domains, all transcripts with predicted ORFs were used for searching conserved functional domains against Pfam database by HMMER tool (version 3.1b2) at an E-value <1e^−5^. Signal peptide and transmembrane domain prediction were performed by signalP (version 4.1) and TMHMM server (version 2.0) tools, respectively. The assignment of Gene Ontology (GO) terms to transcripts were performed based on UniProtKB/SwissProt database to assign unigenes to functional categories. Moreover, online KEGG Automatic Annotation Server (KAAS; http://www.genome.jp/kegg/kaas/) was used to assign pathway information to the transcripts based on 38 plant species and single-directional best hit (SBH) method.

### Differential expression analysis

RSEM tool (http://deweylab.biostat.wisc.edu/RSEM)^70^ was applied to estimate the expression level of transcripts. Initially, each of the cDNA libraries was separately aligned to the reference transcriptome using the Bowtie 2.0^71^. Then, the expression level of each transcript was normalized based on the library size (sequencing depth) and transcripts length and reported in fragments per kb per million (FPKM). To summarize the results and provide statistical tests for pairwise comparisons, the differential expression analysis was performed using edgeR package^72^ (FDR = 0.01 and log_2_^FC^ = 1). Principal component analysis (PCA) was performed to assess the correlation between replicates and treatments. As well, to identify differentially expressed transcription factors (TFs), the homology search was performed against the plant TFs database (PlantTFDB-all_TF_pep) using plantTFDBv4.0 prediction server (http://planttfdb_v4.cbi.pku.edu.cn/prediction.php).

### Gene ontology and KEGG enrichment

To investigate the functional enrichment of the differentially expressed transcripts and visualize the results, the GOseq^73^ and GOplot^74^ packages were used, respectively. The pathways annotation of up and down-regulated transcripts was performed by KAAS (http://www.genome.jp/kegg/kaas/)^75^. The significantly over-represented pathways were screened using the GOseq package (FDR < 0.05).

### RNA-seq results validation by qRT-PCR

Four differentially expressed transcripts in RNA-seq experiment were randomly selected for RNA-seq validation by qRT-PCR. The Primer3 software was used to design the specific primer pairs (Supplementary File 11), and Glyceraldehyde 3-phosphate dehydrogenase (*GAPDH*) was used as a reference gene. qRT-PCR was done using Maxima SYBR Green/ROX qPCR Master Mix (Thermo Scientific). The 15 μl reaction mixture contained 1.0 μl of diluted cDNA sample, 0.5 μl of each forward and reverse primers (10 pM), 10 μl real-time SYBR Green master mix and 3 μl distilled water. The cycling conditions were as initial denaturation at 95 °C for15 min, followed by 40 cycles of 95 °C for 15 s, 60 °C for 30 s and 72 °C for 30 s. Each sample was quantified in three biological replicates. The relative gene expression was quantified by Livack and Schmittgen (2001) method^76^.

## Supplementary information

Supplementary File 1.

Supplementary File 2.

Supplementary File 3.

Supplementary File 4.

Supplementary File 5.

Supplementary File 6.

Supplementary File 7.

Supplementary File 8.

Supplementary File 9.

Supplementary File 10.

Supplementary File 11.

## Data Availability

The datasets generated during and/or analyzed during the current study are available from the corresponding author on reasonable request.
