# Supplementary material for: De novo RNA sequencing analysis of Aeluropus littoralis halophyte plant under salinity stress
Source: Sci Rep. 2020 Jun 4;10:9148. doi: 10.1038/s41598-020-65947-5 (PMC7272644; doi:10.1038/s41598-020-65947-5)
Supplement: Supplementary file 1 [file 41598_2020_65947_MOESM1_ESM.docx]

***De novo* RNA sequencing analysis of *Aeluropus*** ***littoralis* halophyte plant under salinity stress**

Elham Younesi-Melerdi^1^, Ghorban-Ali Nematzadeh^2^, Ali Pakdin-Parizi^1*^, Mohammad-Reza Bakhtiarizadeh^3^ and Seyed-Abolfazl Motahari^4^

^1^ Genetics and Agricultural Biotechnology Institute of Tabarestan, Sari Agricultural Sciences and Natural Resources University, Sari, Iran.

^2^ Department of Agronomy, Sari Agricultural Sciences and Natural Resources University, Sari, Iran.

^3^ Department of Animal and Poultry Science, College of Aburaihan, University of Tehran, Pakdasht, Iran.

^4^ Department of Computer Engineering, [Sharif University of Technology](http://sharif.edu/), Tehran, Iran.

***Corresponding author:**

Dr. Ali Pakdin-Parizi, Genetics and Agricultural Biotechnology Institute of Tabarestan,

Sari Agricultural Sciences and Natural Resources University,

P.O.BOX 578, Sari, Iran, Tel.: +98 11 33687744, Fax: +98 11 33687747,

Email: [a.pakdin@sanru.ac.ir](mailto:a.pakdin@sanru.ac.ir)

**Figure S1.** Results of BUSCO annotations. The dark blue color shows complete and single copies, the light blue color shows complete and duplicate copies, the green color shows the fragmented, and the violet color shows the frequency of non-orthologous transcripts.

**Figure S2.** Contigs length distribution of *A. littoralis* transcriptome.

**Figure S3.** Similarity frequency distribution of different species with the transcriptome of the *A. littoralis*. The BLASTx was performed against the trEMBL plants database.


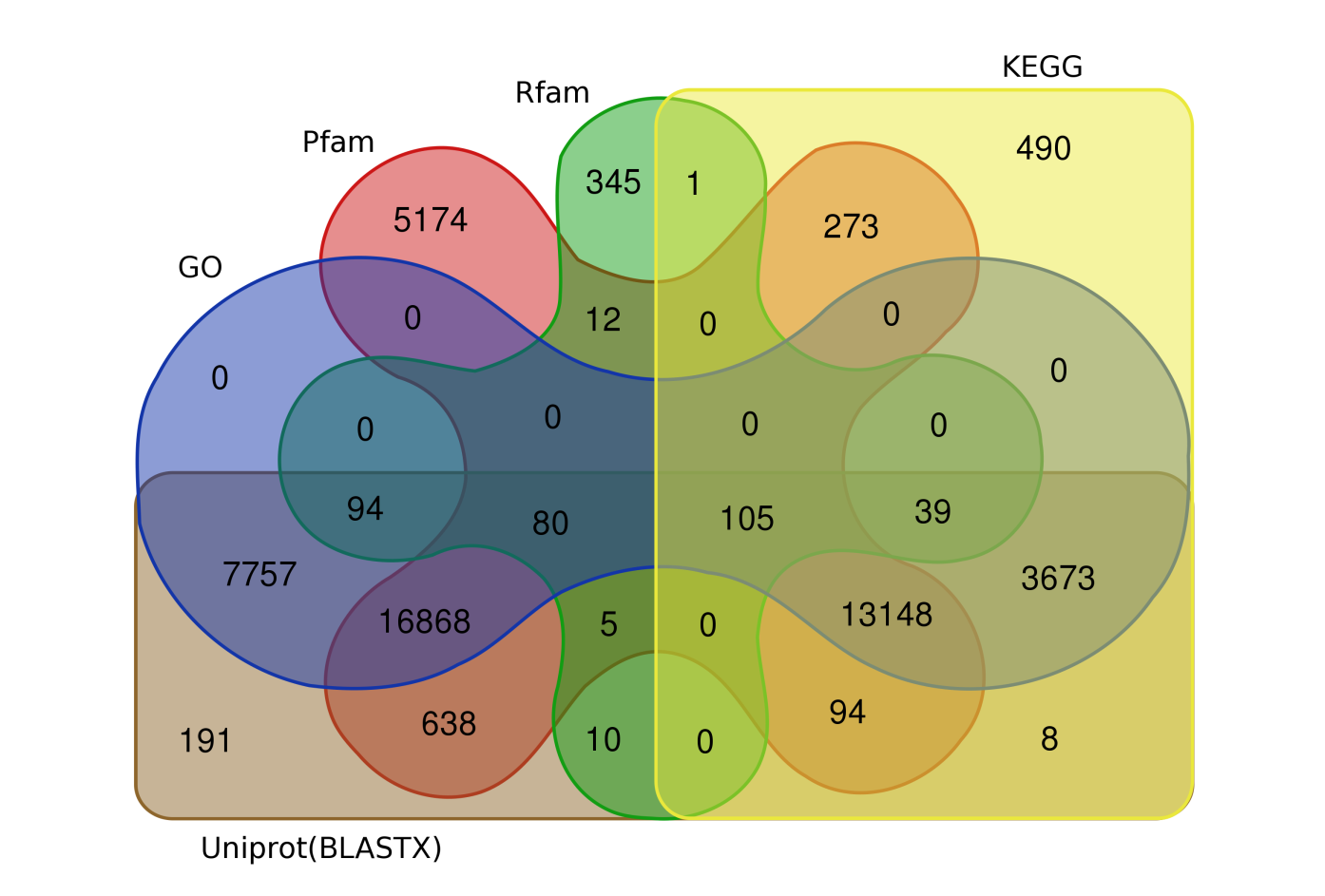


**Figure S4.** The Venn diagram of overlapping annotated transcripts in *A. littoralis* transcriptome against different databases (Pfam, Rfam, KEGG, GO and Uniprot).
