# Supplementary material for: De novo RNA sequencing analysis of Aeluropus littoralis halophyte plant under salinity stress
Source: Sci Rep. 2020 Jun 4;10:9148. doi: 10.1038/s41598-020-65947-5 (PMC7272644; doi:10.1038/s41598-020-65947-5)
Supplement: Supplementary file 4 [file 41598_2020_65947_MOESM4_ESM.pdf]

De novo RNA sequencing analysis of *Aeluropus littoralis* halophyte plant under salinity stress

Elham Younesi-Melerdi[1], Ghorban-Ali Nematzadeh[2], Ali Pakdin-Parizi[1*], Mohammad-Reza Bakhtiarizadeh[3] and Seyed-Abolfazl Motahari[4]

[1] Genetics and Agricultural Biotechnology Institute of Tabarestan, Sari Agricultural Sciences and Natural Resources University, Sari, Iran.

[2] Department of Agronomy, Sari Agricultural Sciences and Natural Resources University, Sari, Iran.

[3] Department of Animal and Poultry Science, College of Aburaihan, University of Tehran, Pakdasht, Iran.

[4] Department of Computer Engineering, Sharif University of Technology, Tehran, Iran.

***Corresponding author:**

Dr. Ali Pakdin-Parizi, Genetics and Agricultural Biotechnology Institute of Tabarestan,
Sari Agricultural Sciences and Natural Resources University,
P.O.BOX 578, Sari, Iran, Tel.: +98 11 33687744, Fax: +98 11 33687747,
Email: a.pakdin@sanru.ac.ir

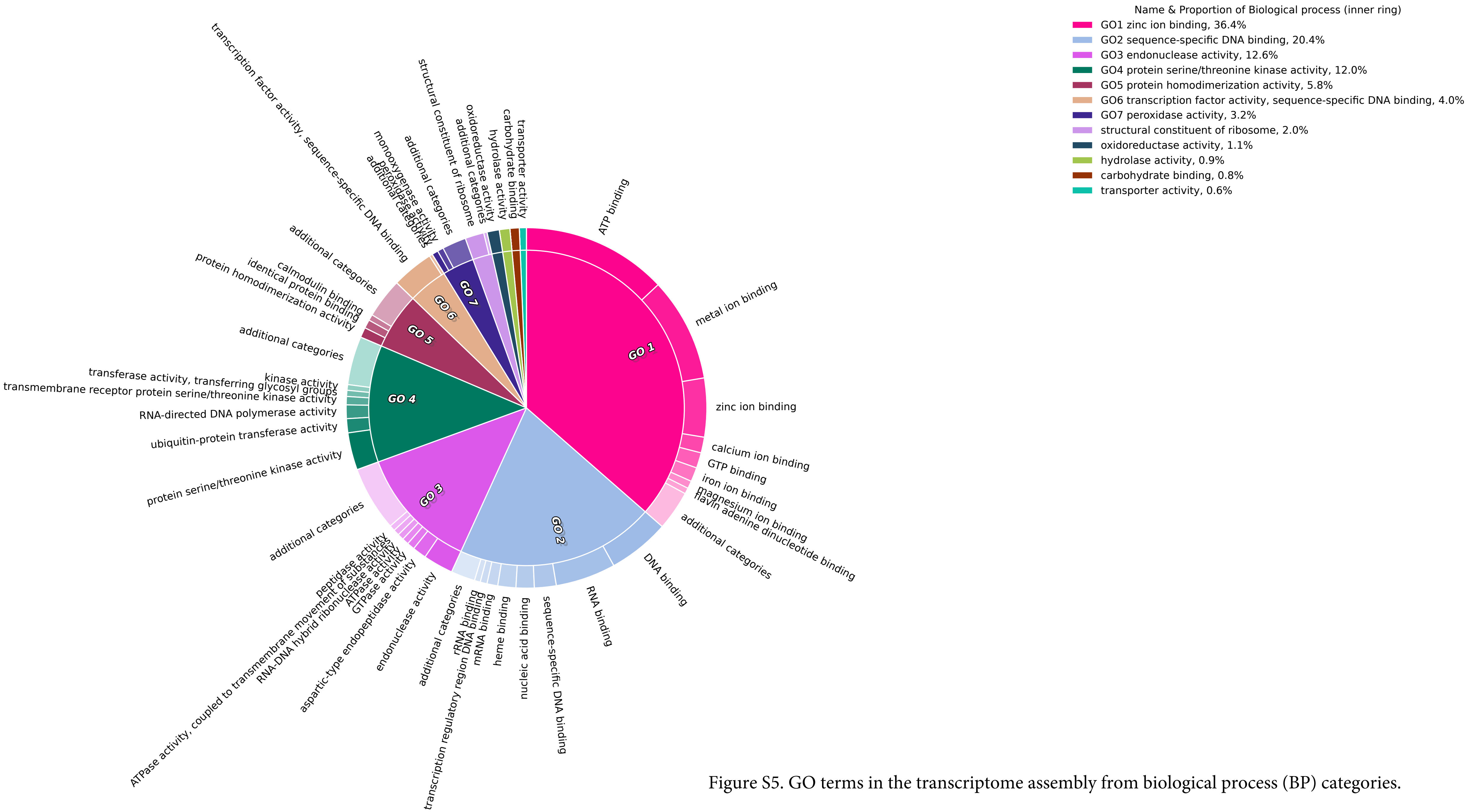

Figure S5. GO terms in the transcriptome assembly from biological process (BP) categories.
